# Characterization of the *Candida albicans* Amino Acid Permease Family: Gap2 Is the Only General Amino Acid Permease and Gap4 Is an *S*-Adenosylmethionine (SAM) Transporter Required for SAM-Induced Morphogenesis

**DOI:** 10.1128/mSphere.00284-16

**Published:** 2016-12-21

**Authors:** Lucie Kraidlova, Sanne Schrevens, Hélène Tournu, Griet Van Zeebroeck, Hana Sychrova, Patrick Van Dijck

**Affiliations:** a VIB Department of Molecular Microbiology, KU Leuven, Flanders, Belgium; b Laboratory of Molecular Cell Biology, Institute of Botany and Microbiology, KU Leuven, Leuven, Belgium; c Department of Membrane Transport, Institute of Physiology, Czech Academy of Sciences, Prague, Czech Republic; University College Dublin, Belfield

**Keywords:** *Candida albicans*, *GAP1*, *S*-adenosyl methionine, general amino acid permease, morphogenesis

## Abstract

Amino acids are key sources of nitrogen for growth of *Candida albicans*. In order to detect and take up these amino acids from a broad range of different and changing nitrogen sources inside the host, this fungus must be able to adapt via its expression of genes for amino acid uptake and further metabolism. We analyzed six *C. albicans* putative general amino acid permeases based on their homology to the *Saccharomyces cerevisiae* Gap1 general amino acid permease. We generated single- and multiple-deletion strains and found that, based on growth assays and transcriptional or posttranscriptional regulation, Gap2 is the functional orthologue to *Sc*Gap1, with broad substrate specificity. Expression analysis showed that expression of all *GAP* genes is under control of the Csy1 amino acid sensor, which is different from the situation in *S. cerevisiae*, where the expression of* ScGAP1* is not regulated by Ssy1. We show that Gap4 is the functional orthologue of *Sc*Sam3, the only *S*-adenosylmethionine (SAM) transporter in *S. cerevisiae*, and we report that Gap4 is required for SAM-induced morphogenesis.

**IMPORTANCE** *Candida albicans* is a commensal organism that can thrive in many niches in its human host. The environmental conditions at these different niches differ quite a bit, and this fungus must be able to sense these changes and adapt its metabolism to them. Apart from glucose and other sugars, the uptake of amino acids is very important. This is underscored by the fact that the *C. albicans* genome encodes 6 orthologues of the *Saccharomyces. cerevisiae* general amino acid permease Gap1 and many other amino acid transporters. In this work, we characterize these six permeases and we show that *C. albicans* Gap2 is the functional orthologue of *Sc*Gap1 and that *C. albicans* Gap4 is an orthologue of *Sc*Sam3, an *S*-adenosylmethionine (SAM) transporter. Furthermore, we show that Gap4 is required for SAM-induced morphogenesis, an important virulence factor of *C. albicans*.

## Abstract (precis)

*Candida albicans* is a commensal organism that can thrive in many niches in its human host. The environmental conditions at these different niches differ quite a bit, and this fungus must be able to sense these changes and adapt its metabolism to them. Apart from glucose and other sugars, the uptake of amino acids is very important. This is underscored by the fact that the *C. albicans* genome encodes 6 orthologues of the *Saccharomyces. cerevisiae* general amino acid permease Gap1 and many other amino acid transporters. In this work, we characterize these six permeases and we show that *C. albicans* Gap2 is the functional orthologue of *Sc*Gap1 and that *C. albicans* Gap4 is an orthologue of *Sc*Sam3, an *S*-adenosylmethionine (SAM) transporter. Furthermore, we show that Gap4 is required for SAM-induced morphogenesis, an important virulence factor of *C. albicans*.

## INTRODUCTION

Over the last 20 years, there has been a noticeable increase in life-threatening fungal infections. The majority of these infections are caused by *Candida* species, mainly *C. albicans*. This fungus typically exists as a harmless commensal in the microflora of the skin, oral cavity, and in the gastrointestinal and urogenital tracts of most humans and other warm-blooded animals. It can be detected in up to 70% of the healthy population (depending on the method of sample collection and the body site) (1, 2). However, *C. albicans* frequently causes oral and vaginal infections (thrush) if the balance of the normal microflora is disrupted by antibiotic usage or when the immune defenses are compromised, such as in HIV patients or in neonates (3, 4). At the most serious level, severely immunocompromised individuals can develop life-threatening systemic infections, such as in the kidney, liver, spleen, or brain (5). Despite the availability of specialized antifungal drugs, such as azoles, polyenes, and echinocandins, mortality rates remain high (6). *C. albicans* is able to infect nearly every site of the human body and has evolved with quick adaptation to the changing availability of nutrients. *C. albicans* is able to utilize various fermentable and nonfermentable carbon sources. In the absence of glucose, this fungus can even use fatty acids or amino acids as a source of carbon (7, 8). Moreover, a diverse range of nitrogen sources can be used by this fungus. Some of them are used preferentially (ammonia, glutamine, asparagine, and glutamate); however, when these primary nitrogen sources are limited or not available, *C. albicans* can also use less-preferred nitrogen sources, like isoleucine, tyrosine, and tryptophan (9). In the host, the largest proportion of amino acids is fixed in host proteins, requiring the production of proteolytic enzymes. *C. albicans* produces several proteases, all belonging to the secreted aspartyl proteinase (SAP) gene family (10). They are independently regulated and functionally distinct and allow the fungus to break down or decompose almost every tissue of the host into suitable nutrients (11, 12).

In the model yeast *Saccharomyces cerevisiae*, amino acid uptake is mediated by a family of 20 amino acid permeases. Most of them are specific for one or a few related l-amino acids. One exception is *Sc*Gap1, which has broad substrate specificity, high capacity, and mediates the uptake of most l- and d-amino acids, nonproteic amino acids including citrulline and ornithine, and a number of toxic amino acid analogues (13). The induction of its expression as well as the activity of *Sc*Gap1 are tightly regulated according to both the quality and quantity of the nitrogen sources present in the medium. The presence of preferred nitrogen sources (ammonium, glutamate, and glutamine) or of high amino acid levels represses *ScGAP1* expression and inactivates the transporter by ubiquitination and internalization for targeting to the vacuole and subsequent degradation (14–16). The transcription of *ScGAP1* is regulated by the nitrogen catabolite repression (NCR) pathway (17). When cells are growing in medium with poor nitrogen sources, e.g., proline or urea, *ScGAP1* expression is activated by two GATA-type transcription factors, and neosynthesized *Sc*Gap1 accumulates at the plasma membrane in an active, stable form (18).

In addition to its transport function, *Sc*Gap1 was found to play a role as an amino acid sensor for the rapid activation of protein kinase A (PKA) upon addition of amino acids to nitrogen-starved cells (19, 20). Little is known about transport and signaling properties of Gap permeases in *C. albicans* or about their regulation. Phylogenetic analysis shows that the *C. albicans* genome encodes six orthologues of *ScGAP1* (21). Based on findings with complementation experiments in *S. cerevisiae* amino acid transporter mutants, we previously showed that Gap2 is the functional *Sc*Gap1 orthologue and that the others function as more specific permeases (21). We also showed that Gap1, Gap2, and Gap6 function not only as amino acid transporters but also, similarly to *Sc*Gap1, as amino acid sensors for activation of the PKA pathway. Nevertheless, all these results were obtained by expressing the *C. albicans* genes in *S. cerevisiae* mutants lacking their own amino acid transporters.

Apart from *Sc*Gap1, another plasma membrane sensor for extracellular amino acids has been described in *S. cerevisiae*, Ssy1. It is a unique member of the amino acid permease family which is not able to transport amino acids but controls amino acid uptake in exponentially growing cells by inducing the transcription of genes encoding several amino acid permeases, such as *Sc*Dip5 and *Sc*Agp1, but not *Sc*Gap1 (22–24). In *C. albicans*, it was shown that the expression of *GAP1* and *GAP2* is under control of the amino acid sensor Csy1, which is an orthologue of Ssy1 (25). Whereas this is different from the situation with *S. cerevisiae*, another level of *GAP2* regulation which entails upregulation during nitrogen starvation and repression upon subsequent addition of arginine to the cells is similar to what is observed in *S. cerevisiae* for *ScGAP1* (26).

In this study, we systematically analyzed all six members of the *C. albicans* general amino acid permease family, and we confirmed Gap2 as the only and real *Sc*Gap1 general amino acid permease orthologue, based on its broad substrate specificity and for its similar transcriptional regulation. Moreover, we show that all *GAP* genes are under control of the Csy1 amino acid sensor, which is different from the situation in *S. cerevisiae*. In addition to this, we also show that Gap4 is the orthologue of the *S. cerevisiae* Sam3 transporter, as Gap4 is required for *S*-adenosylmethionine (SAM) transport and SAM-induced morphogenesis.

## RESULTS

### The *C. albicans* genome encodes six *Sc*Gap1 orthologues*.*

**FIG 1:**
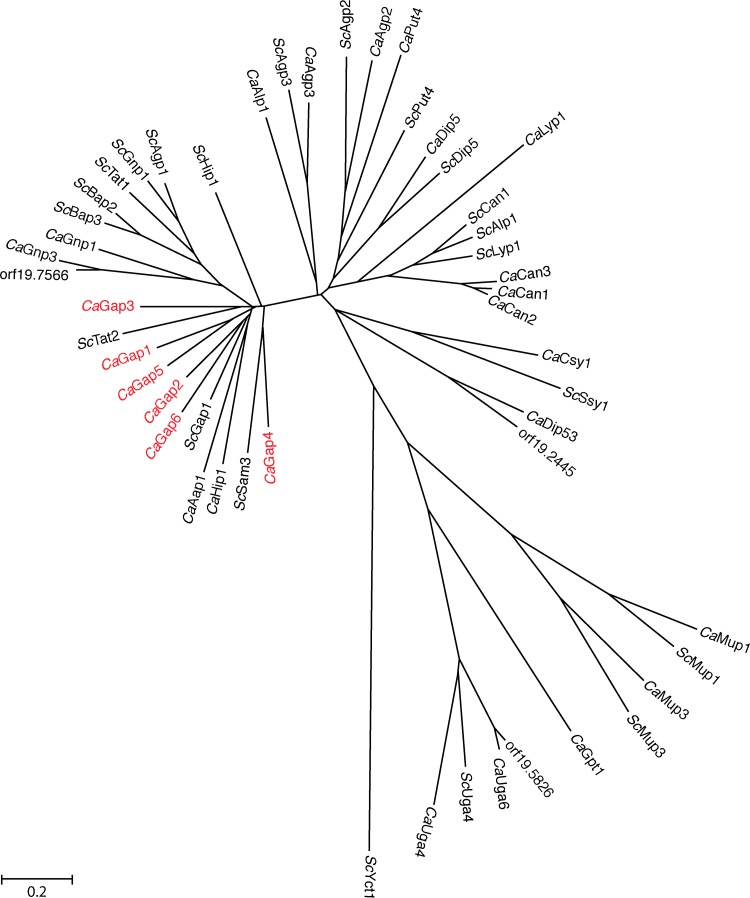
Phylogenetic tree of amino acid permeases. All known amino acid permeases in *S. cerevisiae*, the six-member *C. albicans* Gap family, and all other putative amino acid permeases in *C. albicans* were analyzed using the software package MEGA (version 4). Distances between the permeases are related to the degree of divergence between sequences. Evolutionary distances were computed using the maximum composite likelihood method. The scale bar indicates 0.2 amino acid substitutions per site.

A BLAST analysis with the *Sc*Gap1 protein sequence as a query against the two available *C. albicans* databases, CGD (27) and CandidaDB (28), resulted in the identification of six putative orthologues (21). A reciprocal BLAST analysis of these six putative Gap protein sequences against the *S. cerevisiae* SGD database (29) always resulted in the *Sc*Gap1 permease as the closest orthologue (21). Phylogenetic analysis of the six *C. albicans* Gap protein sequences as well as all the other *C. albicans* amino acid permease protein sequences with all the *S. cerevisiae* amino acid permease protein sequences showed that all Gap permeases belong to the same cluster as *Sc*Gap1, a cluster that also includes *Sc*Hip1 (histidine permease) and *Sc*Tat2 (tryptophan/tyrosine permease) (Fig. 1). Gap2 constitutes the closest *Sc*Gap1 orthologue, while Gap4 is the most distantly related permease in the phylogenetic tree. Using Gap4 as a query, we found *Sc*Sam3 was the second best orthologue. *Sc*Sam3 is the high-affinity permease that mediates transport of SAM across the plasma membrane of *S. cerevisiae* (30). While we have characterized all six genes from the *GAP* gene family, these *in silico* results prompted us to focus mainly on Gap2 and Gap4. Gene names and systematic names of all the *CaGAP* genes are listed in Table 1.

**TABLE 1:** *CaGAP* genes used in this study

Gene name	Systematic name^a^	Length (bp)
*CaGAP1*	orf19.11780	1,749
*CaGAP2*	orf19.6993	1,767
*CaGAP3*	orf19.10706	1,800
*CaGAP4*	orf19.11936	1,824
*CaGAP5*	orf19.9365	1,857
*CaGAP6*	orf19.6659	1,707

a According to the Candida Genome Database.

### *GAP2* is the only orthologue of *ScGAP1*, but important differences with the *S. cerevisiae* protein exist. (i) *GAP2* has broad substrate specificity.

We generated *GAP* single-deletion strains by using the fusion PCR method (31, 32). Previous results showed that several of the permeases have overlapping amino acid transport capacities (21). Therefore, we also generated double and triple *GAP* deletion strains, *gap1Δ/gap1Δ gap2Δ/gap2Δ*, *gap2Δ/gap2Δ gap6Δ/gap6Δ*, and *gap1Δ/gap1Δ gap6Δ/gap6Δ*, and also *gap1Δ/gap1Δ gap2Δ/gap2Δ gap6Δ/gap6Δ*, using the *SAT1* flipper cassette (33, 34). The triple-deletion strain was generated because the three permeases can each transport leucine, methionine, and phenylalanine (21). All strains were confirmed by PCR analysis and Southern blotting (data not shown).

**FIG 2:**
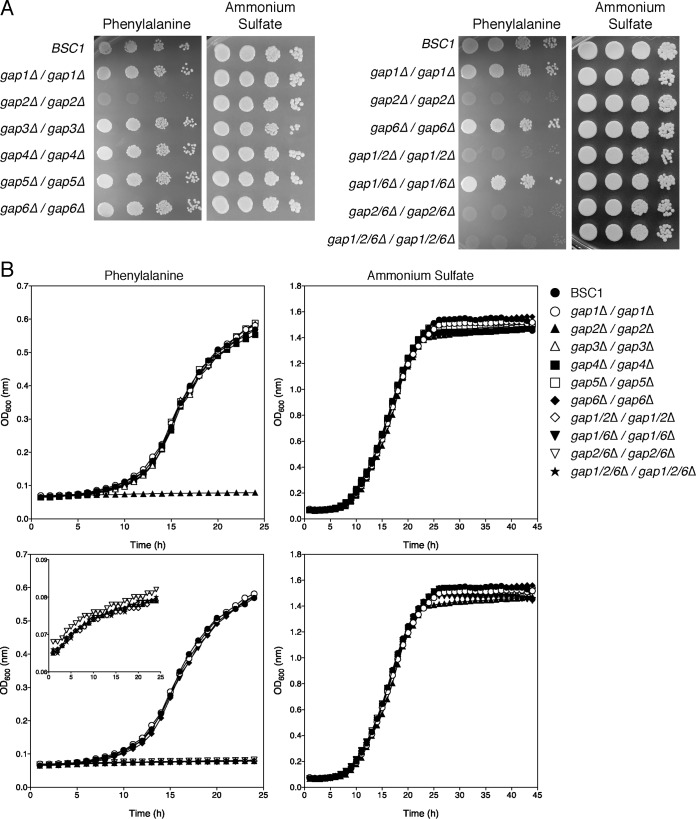
Gap2 is required for growth on phenylalanine as the sole source of nitrogen. Cells from an overnight grown culture on YPD plates were resuspended and diluted to an OD_600_ of 1 for drop tests or to an OD_600_ of 0.002 for liquid growth assays. (A) Three microliters of 10-fold dilutions were spotted on YNB medium with phenylalanine (300 µg/ml) or ammonium sulfate (5 mg/ml) as the sole source of nitrogen. Pictures were taken after 2 days. (B) For the liquid growth assays, YNB medium (supplemented with 300 µg/ml of phenylalanine or ammonium sulfate [5 mg/ml] as the sole source of nitrogen) was used, and 100-µl aliquots were incubated at 30°C in 96-well microtiter plates in an absorbance microplate reader. The optical density in each well was measured over a period of 24 h. All strains that had a deletion in *GAP2* were unable to grow in medium containing phenylalanine as the sole source of nitrogen. The inset in the lower left panel shows the absence of growth (OD from 0.07 to 0.08 over a period of 24 h).

To determine whether any of the mutant strains had a defect in the uptake of amino acids, we tested growth for all of them on yeast nitrogen base (YNB) agar plates containing 300 µg/ml of one single specific amino acid. As a positive control, we used wild-type strain SN87 that had been made prototrophic for leucine and histidine (strain BSC1). We tested all amino acids that showed a growth phenotype for the *S. cerevisiae* mutant strains expressing single *GAP* genes (21). Only the *gap2Δ/gap2Δ* strain showed a growth defect on medium containing phenylalanine (Fig. 2A, left panels) or valine, leucine, or methionine (see Fig. S1 in the supplemental material) as sole nitrogen source. As a general control for growth, we used YNB plus 300 µg/ml of ammonium sulfate plates (Fig. 2A, right panels).

10.1128/mSphere.00284-16.1 Figure S1  Utilization of specific amino acids as the sole source of nitrogen by the different *GAP* deletion strains. Cells from an overnight grown culture on YPD plates were resuspended and diluted to an OD_600_ of 1 for drop tests or to an OD_600_ of 0.002 for liquid growth assays. (A) Three-microliter aliquots of 10-fold dilutions were spotted on YNB medium with the indicated amino acids (at 300 µg/ml) as the sole source of nitrogen. Pictures were taken after 2 days. (B) For the liquid growth assays, YNB medium (supplemented with 300 µg/ml of the specific amino acids as the sole sources of nitrogen) was used, and 100-µl aliquots were grown at 30°C in 96-well microtiter plates in an absorbance microplate reader, and the optical density in each well was measured over a period of 24 h. Download Figure S1, TIF file, 1.4 MB. Copyright © 2016 Kraidlova et al. 2016 Kraidlova et al. This content is distributed under the terms of the Creative Commons Attribution 4.0 International license.

Despite the clear growth defect of the *gap2Δ/gap2Δ* strain, growth was not completely abolished on medium with phenylalanine as the N source. This finding may point to the ability of other transporters to take up the tested amino acid. We therefore tested the involvement of Gap1 and Gap6, which we previously showed that, upon expression in *S. cerevisiae*, are able to take up methionine, leucine, and phenylalanine (21). However, additional deletion of *GAP1* or/and *GAP6* did not further decrease the capacity to grow on single amino acids as the sole source of nitrogen (Fig. 2A, left panels for phenylalanine; see Fig. S1 for results with valine, leucine, and methionine).

Similar results were obtained in liquid media (Fig. 2B). Growth assays in the presence of YNB supplemented with specific amino acids showed that Gap2 is required for growth on phenylalanine (Fig. 2B, left panels) and on methionine, valine, leucine, tyrosine, tryptophan or lysine (Fig. S1) as sole source of nitrogen. On the other hand, Gap2 is not required for growth on medium containing ammonium sulfate (Fig. 2B, right panels), glutamine, arginine, or proline (Fig. S1) as the sole source of nitrogen. These results showed that Gap2 is the only amino acid permease with very broad substrate specificity. None of the other Gap permeases was able to transport (based on growth assays) such a variety of amino acids (21). Reintegration of *GAP2* in the *gap2Δ/gap2Δ* strain restored growth on media containing specific amino acids (data not shown), hence linking the phenotype to the absence of Gap2.

### (ii) In contrast to *S. cerevisiae*, in which citrulline is only transported by *Sc*Gap1, several amino acid permeases exist in *C. albicans* that are able to transport citrulline.

**FIG 3:**
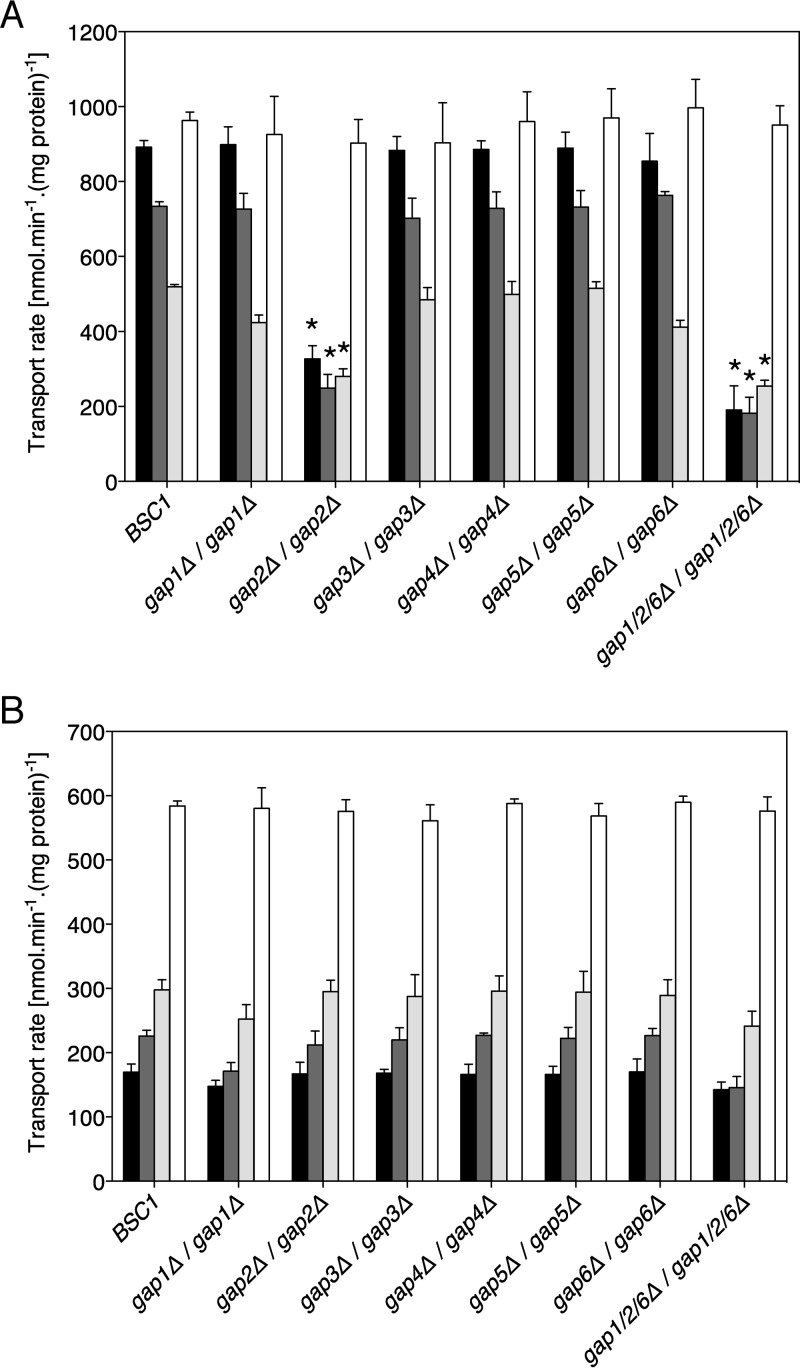
Uptake of amino acids by *C. albicans*. The wild type and strains lacking one of the *GAP* gene family members or lacking 3 *GAP* genes (*GAP1*, *GAP2*, and *GAP6*) were used. Transport of 2 mM citrulline (black bars), leucine (dark gray bars), phenylalanine (light gray bars), or glutamine (white bars) was measured using cells which were incubated under nitrogen starvation conditions (without any source of nitrogen) (A) or growing under nitrogen-rich conditions (with ammonium sulfate as nitrogen source) (B). The level of transport is expressed as nanomoles of amino acid transported per minute per milligram of protein, and statistical significance (*) was determined by comparison with the wild type.

Citrulline is an amino acid that has been used frequently in studies of *S. cerevisiae*’s transport and signaling capacities via *Sc*Gap1. At concentrations lower than 5 mM, citrulline can only be transported appreciably by *Sc*Gap1 (13, 35). It was previously described that deletion of *GAP1* has an effect on citrulline uptake in *C. albicans* under nitrogen-poor conditions (36). In our hands, however, a single deletion of the *GAP1* gene, or single deletions of either *GAP3*, *GAP4*, *GAP5*, or *GAP6*, had no effect on citrulline uptake under nitrogen-poor conditions, whereas citrulline uptake was significantly lower in the *gap2Δ/gap2Δ* strain than in the wild type (*P* < 0.001) (Fig. 3A). Citrulline transport was even more reduced in the triple-deletion mutant, *gap1Δ/gap1Δ gap2Δ/gap2Δ gap6Δ/gap6Δ*, than in the single-deletion *gap2/gap2Δ* mutant. Since 20% of citrulline transport remained in the triple-deletion strain, other transporters for citrulline must be present in *C. albicans*.

To confirm the growth experiment results with different amino acids, we also measured the rate of uptake of 2 mM leucine, phenylalanine, and glutamine. Whereas uptake of leucine and phenylalanine showed similar trends as uptake of citrulline (significant differences between the wild-type and *gap2Δ/gap2Δ* or *gap1Δ/gap1Δ gap2Δ/gap2Δ gap6Δ/gap6Δ* strains), none of the tested strains showed differences in glutamine uptake, which was in accordance with the results of the growth experiments (Fig. S1).

In *S. cerevisiae*, the general amino acid permease system is strongly repressed under nitrogen-rich conditions (37). To investigate this in *C. albicans*, we determined citrulline, leucine, phenylalanine, and glutamine uptake in cells grown in the presence of ammonium sulfate. Gap2 was clearly not involved in amino acid uptake under these conditions, pointing to repression under these conditions (see below). Surprisingly, the *gap1Δ/gap1Δ* strain and the *gap1Δ/gap1Δ gap2Δ/gap2Δ gap6Δ/gap6Δ* triple mutant exhibited mildly decreased uptake of citrulline, leucine, and phenylalanine (Fig. 3B). For glutamine, we could not observe any difference between the BSC1 control strain and any of the tested strains. In summary, the uptake measurements confirmed the results obtained in the growth assays and clearly showed that only Gap2 can be considered a functional orthologue of *Sc*Gap1. It is also interesting that the repression of Gap2 transport activity in nitrogen-containing medium almost mimics the effect of a deletion of this gene.

### (iii) Expression of all *CaGAP* genes is under control of the Csy1 amino acid sensor.

**FIG 4:**
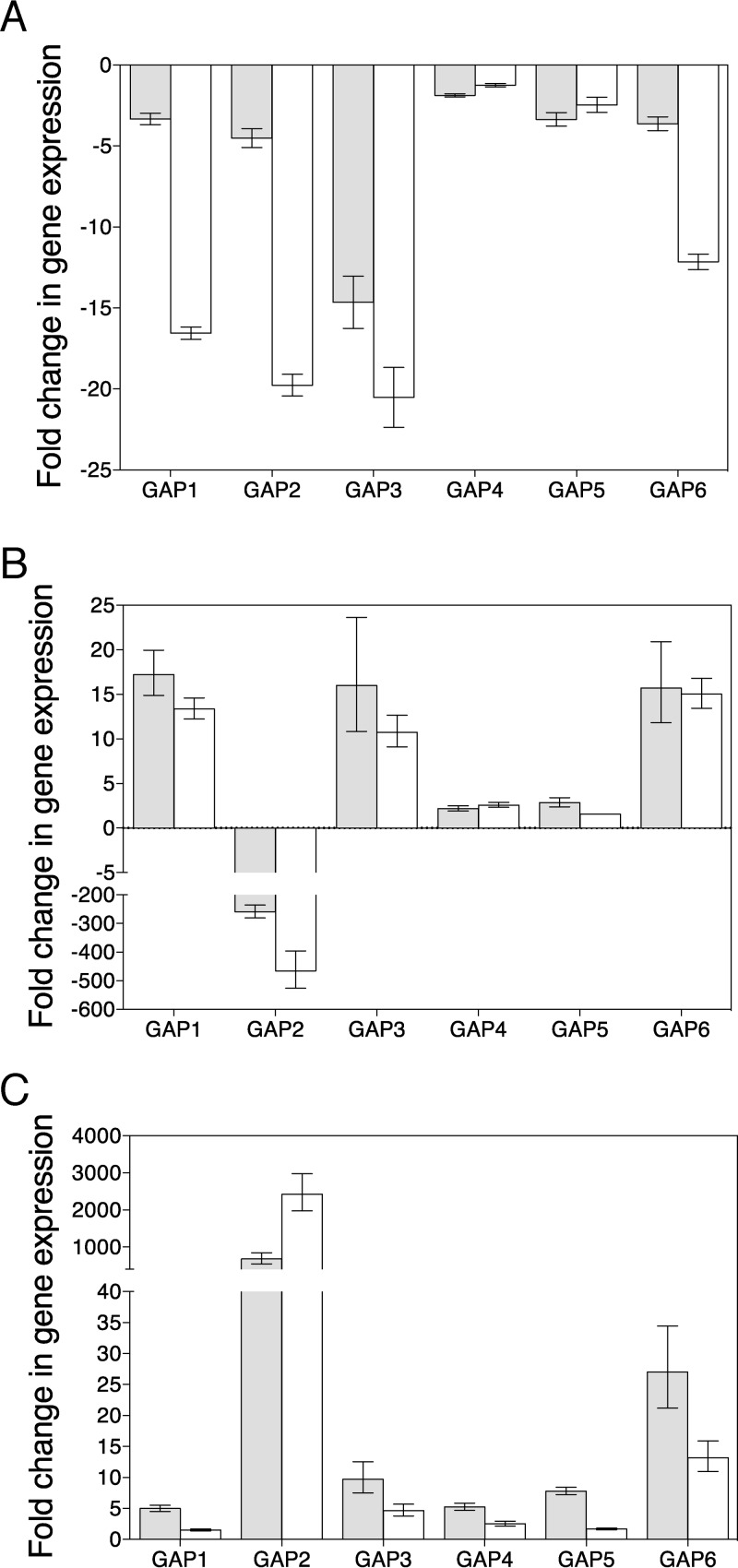
Expression of all the *GAP* genes is under control of the Csy1 sensor and regulated by nitrogen sources. (A) Expression of the six *GAP* genes was analyzed in the BSC1 (wild type) and the *csy1* (CAEB-5) mutant strain. Nitrogen-starved cells were resuspended in YNB medium supplemented with 5 mg/ml of ammonium sulfate supplemented with 100 µg/ml histidine, and samples were taken after 15 (gray bars) or 30 min (white bars). The fold change in gene expression of the *GAP* genes in the *csy1* mutant relative to expression in the wild-type strain is shown. (B) The fold changes in gene expression levels between *GAP* genes when cells were shifted from nitrogen starvation medium to YPD medium. (C) Fold changes in gene expression levels between *GAP* genes when cells were shifted from YPD medium to nitrogen starvation medium. Gray bars show expression at 15 min and white bars show levels at 30 min, on the basis of shifting to the new medium (for panels B and C). The comparative *C*_*T*_ method to calculate relative changes in gene expression was used, with *TEF1* as the housekeeping gene. Error bars show the range of possible relative quantity values defined by the standard errors of the ΔΔ*C*_*T*_ values. All experiments were evaluated for at least three RNA preparations.

*S. cerevisiae* and *C. albicans* each express an amino acid transporter homologue, Ssy1 and Csy1, respectively, which in both cases lost the capacity to transport. However, in the presence of histidine, they function as sensors to detect amino acids in the medium, resulting in the induction of specific amino acid transporter genes (23, 25). The specific transporter genes controlled by this sensor are different between *S. cerevisiae* and *C. albicans*. *ScGAP1* expression is not regulated by Ssy1. Previous gene expression analysis in *C. albicans* showed that both *GAP1* and *GAP2* are under control of Csy1 (25). Here, we confirmed this and showed that the expression of all *GAP* genes is under control of Csy1 (Fig. 4A). This is one of the main regulatory differences between *GAP2* and *ScGAP1*. We observed slightly different regulation for *GAP4* and *GAP5* compared to the others, as the fold change difference was bigger after 15 min than after 30 min, whereas this was clearly not the case for the other *GAP* genes.

### (iv) Expression of *CaGAP2* is regulated by nitrogen sources, similar to *ScGAP1*.

We performed quantitative real-time PCR (qRT-PCR) to quantify gene expression of the different *GAP* genes when cells were shifted from medium without any source of nitrogen (nitrogen starvation conditions) to yeast extract-peptose-dextrose (YPD) medium or to YNB medium with ammonium sulfate as nitrogen source (Fig. 4B). Expression of the *GAP2* gene was rapidly and strongly repressed when cells were shifted to YPD, whereas the expression of the other *GAP* genes (mainly *GAP1*, *GAP3*, and *GAP6*) was induced (Fig. 4B). Transfer of the cells from nitrogen starvation medium to YNB with ammonium sulfate led to similar results, only the values were lower for all investigated genes (data not shown).

When performing the opposite shift (from YPD to nitrogen starvation), we observed a very strong induction of the *GAP2* gene (Fig. 4C), whereas the other *GAP* genes were transiently upregulated to a very low extent, except for *GAP6*, which was more strongly upregulated upon a shift to nitrogen starvation (Fig. 4C).

**FIG 5:**
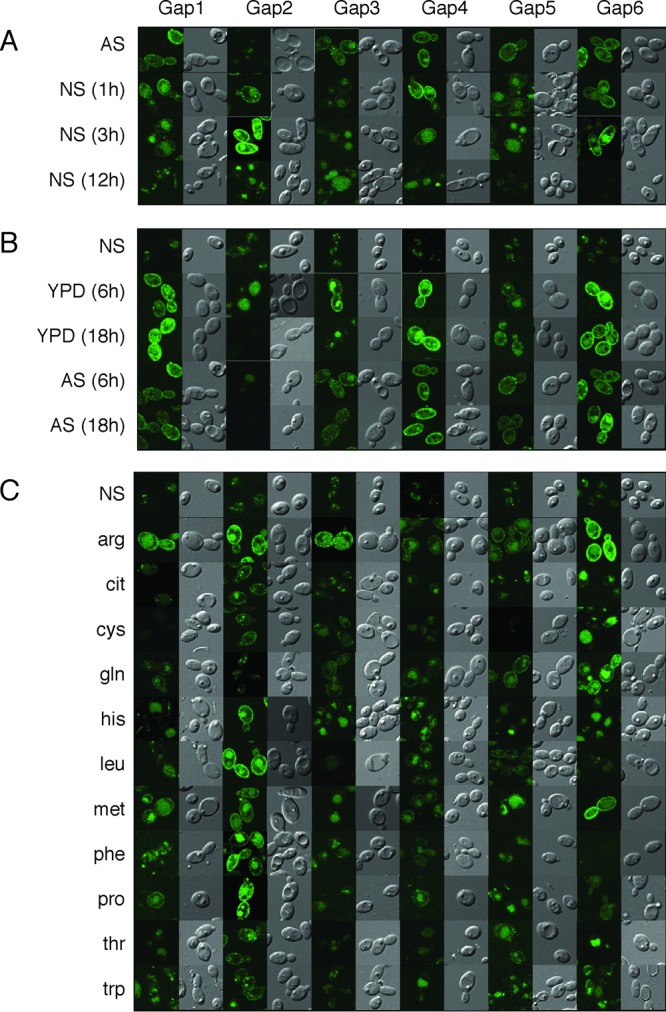
Fluorescence microscopy of Gap-GFP fusion constructs under different environmental conditions. (A) Cells were grown in YNB medium supplemented with 5 mg/ml of ammonium sulfate (AS) to exponential phase and then transferred to nitrogen starvation medium (NS). Fluorescence and DIC (Nomarski) images were taken after 1 h, 3 h, or 12 h. (B) Cells were placed in NS for 12 h and then transferred to YPD or YNB medium supplemented with 5 mg/ml of AS. Fluorescence and DIC (Nomarski) images were taken after 6 h or 18 h. (C) Cells were incubated in nitrogen starvation medium for 12 h and then transferred to YNB medium supplemented with 100 µg/ml of a single amino acid as the sole source of nitrogen. Fluorescence and DIC (Nomarski) images were taken after 5 to 6 h. The pictures shown are representative for all living cells in our experiments.

Apart from transcriptional regulation, *Sc*Gap1 is also posttranslationally regulated. When *Sc*Gap1 is in the plasma membrane (during nitrogen starvation or during growth on nitrogen-poor sources), addition of ammonium, glutamine, or glutamate causes internalization of the *Sc*Gap1 permease (37). To determine the cellular localization and to follow the fate of the different Gap permeases under the different nitrogen source conditions, we generated strains where one of the endogenous *GAP* genes was tagged with the *GFP*. This allowed us to determine the presence of the different Gap proteins and their location in the cell during growth under nitrogen-poor or nitrogen-rich conditions (Fig. 5). The strong induction of *GAP2* when cells were shifted from YPD to nitrogen starvation medium was confirmed with the GFP construct, as clear plasma membrane-localized expression was observed. At later time points (12 h), the permease was no longer in the membrane but seemed to be degraded in the vacuole (Fig. 5A). This was a second major difference with the situation in *S. cerevisiae*, where *Sc*Gap1 remained in the plasma membrane under nitrogen starvation conditions. To follow the expression of the other *GAP* genes, the cells were shifted from YNB with ammonium sulfate to nitrogen starvation medium. Similar to the qRT-PCR data, these permeases were expressed at low levels, and they were all rapidly degraded. We also performed GFP expression analysis for the opposite shift. Cells from nitrogen starvation conditions were transferred to YPD or YNB with ammonium sulfate, and pictures were taken after 6 h and 18 h (Fig. 5B). Gap2 was rapidly degraded, whereas all other Gap proteins (except Gap5, for which expression was not changed) were stably expressed in the plasma membrane, in accordance with the qRT-PCR experimental findings (Fig. 5B). We then studied the expression of the Gap proteins after shifting the cells from nitrogen starvation medium to YNB medium supplemented with a single amino acid as the sole source of nitrogen (Fig. 5C). As was expected from the previous results, the Gap2 protein was present in the plasma membrane under all tested conditions except YNB with ammonium sulfate or glutamine, similar to the findings with *Sc*Gap1. The other investigated proteins were all present in the plasma membrane in medium with ammonium sulfate, arginine, or glutamine, with minor differences in expression patterns. For Gap1, the presence at the plasma membrane was observed also when methionine, phenylalanine, or proline was present in the medium as the sole nitrogen source. For Gap4, this was the case when histidine, leucine, methionine, or proline was present in the medium as the sole source of nitrogen. In addition, while Gap5 was also localized at the plasma membrane when leucine was present in the medium as the sole source of nitrogen, Gap6 was detected when methionine or proline was the sole source of nitrogen.

### Gap4 is involved in SAM transport and is required for SAM-induced morphogenesis.

**FIG 6:**
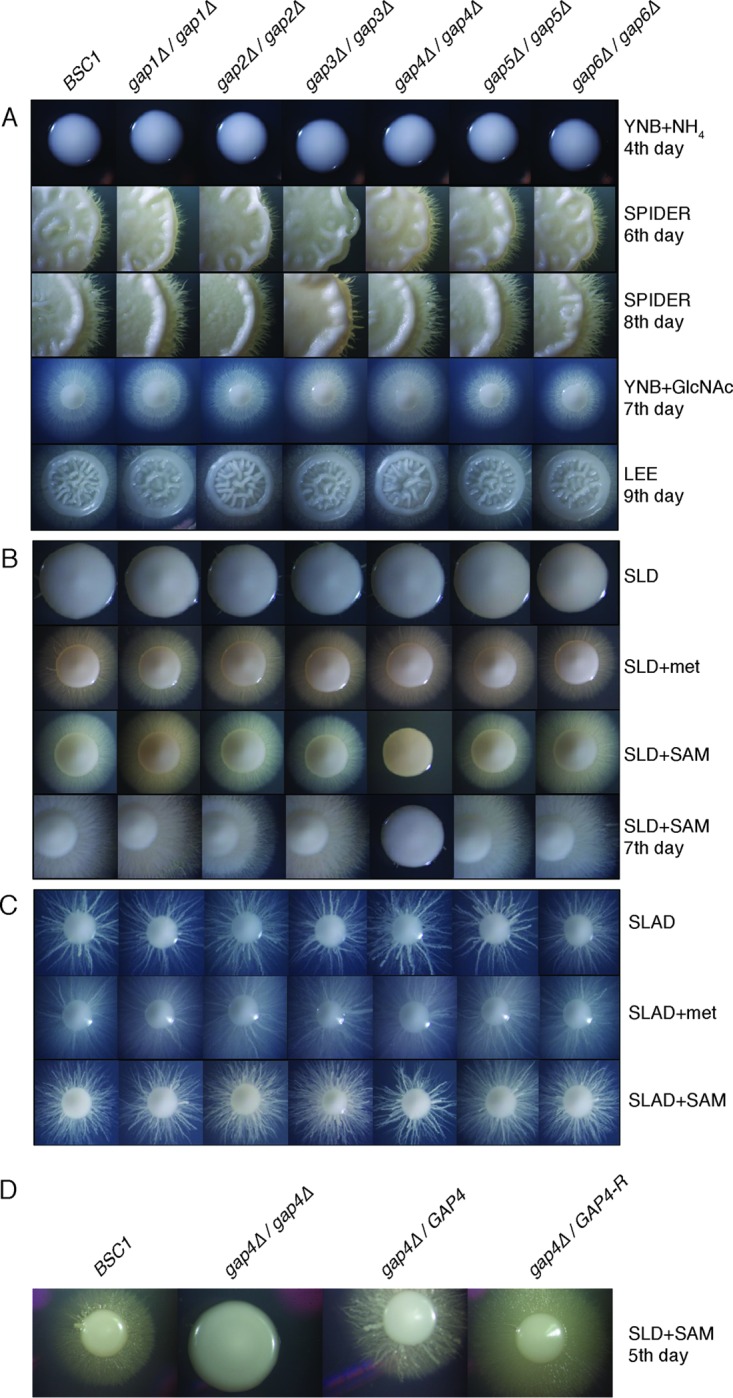
Morphological analysis of the *GAP* deletion mutants under different hypha-inducing conditions. Wild-type and *GAP* deletion strains were grown on YPD plates and then diluted in PBS, and approximately 10 cells were plated on different solid growth media. Time of incubation and temperature were as follows. (A) YNB plus ammonium sulfate, 4 days at 30°C; SPIDER, 6 or 8 days at 37°C; YNB plus GlcNAc, 7 days at 37°C; LEE, 9 days at 30°C. (B) SLD or SLD plus 5 µg/ml methionine, 5 days at 37°C; SLD plus 5 µg/ml SAM, 5 or 7 days at 37°C. (C) SLAD, or SLAD plus 5 µg/ml methionine, or SLAD plus 5 µg/ml SAM, 5 days at 37°C. (D) SLD plus 5 µg/ml SAM, 5 days at 37°C. Pictures of representative colonies were taken using a binocular microscope. Results for the wild-type strain BSC1, the *Cagap* deletion strains, and the *gap4/GAP4*-R reintegrant strain are shown.

We tested the different *gap* mutants for defects/changes in colony morphology and hypha formation. Strains were grown on several solid media known to induce the morphological switch (Spider, SLAD, LEE medium, and medium containing *N*-acetylglucosamine) (38–42) and on SLD and YNB media containing NH_4_ as a control. In addition, we tested the influence of methionine and SAM on colony morphology. On the control media, all our investigated strains showed smooth colonies (Fig. 6A and B). Under hypha-inducing conditions, all strains clearly showed comparable hyphal morphogenesis (Fig. 6A to D), including the *gap1Δ/gap1Δ* mutant, which was previously shown to have a defect in morphogenesis in Spider medium (36). Only the *GAP3* deletion strain was slightly defective in hypha formation in Spider medium; hyphae started to grow 2 days later than in all other deletion and wild-type strains (Fig. 6A). The same phenotype was shown for three independent *gap3Δ/gap3Δ* mutants. The *gap4Δ/gap4Δ* strain showed a strong defect in morphogenesis on SLD plus SAM medium. Colonies of this strain were smooth, and they did not start to create hyphae even after prolonged incubation (7 days) (Fig. 6B).

**FIG 7:**
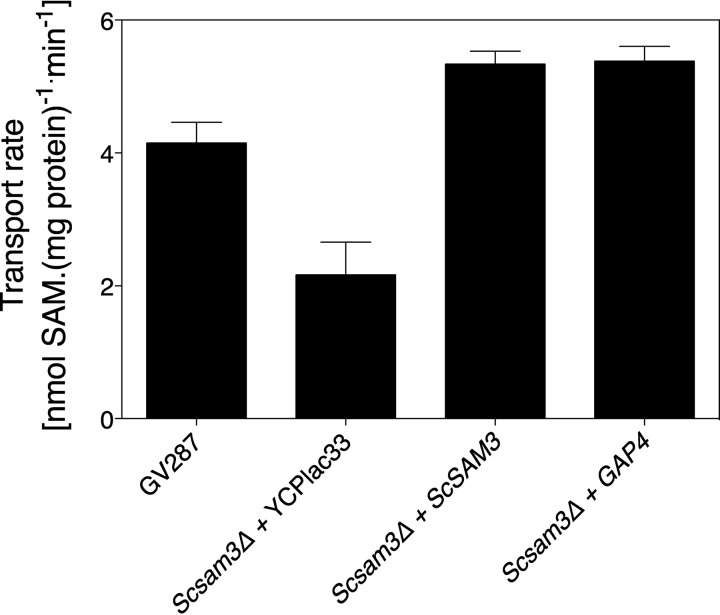
SAM transport by Gap4 in *S. cerevisiae* cells. Transport of SAM in wild-type cells, the *sam3*Δ strain transformed with the empty vector, the *sam3*Δ strain complemented with *ScSAM3*, or the *sam3Δ* strain complemented with *GAP4* is shown. Results of one representative experiment are shown, and the error bars show the standard deviations of three different transformants. All transformants were statistically significantly different from the wild type, and expression of both *SAM3* and *GAP4* showed a statistically significant difference from the strain transformed with the empty plasmid.

As our phylogenetic tree showed that Gap4 is homologous not only to *Sc*Gap1 but also to *Sc*Sam3, the *S. cerevisiae* high-affinity SAM transporter (*K*_*m*_ of 3.3 µM), we hypothesized that Gap4 may be responsible for the transport of this metabolite in *C. albicans* (30). To determine whether Gap4 is a SAM transporter, we expressed the *GAP4* gene in an *S. cerevisiae sam3Δ* strain. The expression was under control of the *SAM3* promoter and terminator sequences, and expression of *SAM3* itself was used as a positive control. SAM transport assays clearly showed that Gap4 is a SAM transporter (Fig. 7; Fig. S2).

10.1128/mSphere.00284-16.2 Figure S2  SAM transport by Gap4. This is an independent repetition of an experiment shown in Fig. 7. The legend of Fig. 7 explains this figure. Download Figure S2, TIF file, 0.2 MB. Copyright © 2016 Kraidlova et al. 2016 Kraidlova et al. This content is distributed under the terms of the Creative Commons Attribution 4.0 International license.

## DISCUSSION

*C. albicans* lives in the human body as a commensal and under specific conditions as a pathogen. Under both conditions, the cells must be able to sense and take up the available nutrients present in a specific niche. To allow the uptake of amino acids, *C. albicans* cells secrete a large number of hydrolase enzymes (mainly belonging to the secreted aspartyl proteinase gene family) that convert proteins into peptides and amino acids that can be taken up by peptide transporters or amino acid permeases, respectively (26, 43). Previously, it was shown that the capacity to take up amino acids is strongly linked with virulence. Deletion of the endoplasmic reticulum-localized packaging chaperone Csh3 results in a defect in the expression of amino acid permeases, and a strain with such a deletion showed virulence defects (44). *C. albicans* expresses 28 putative amino acid transporters and 1 amino acid sensor (Csy1) (21, 25, 27, 45, 46). Phylogenetic analysis of the *C. albicans* permeases indicates that there are six orthologues of the *S. cerevisiae* Gap1, which is the general amino acid permease and is able to transport most amino acids. The fact that there are six *ScGAP1* orthologues underscores the importance of amino acid uptake for this human fungal pathogen. Several more specific amino acid permeases are also expressed under different conditions. We have previously characterized the transport capacities of these six permeases by expressing them in yeast mutant permease strains (21). Gap2 turned out to be the permease with the broadest transport capacities, resembling that of *Sc*Gap1. Here we confirmed, based on growth and transport assay results, that Gap2 can be considered the functional orthologue of *Sc*Gap1. Deletion of only *GAP2* showed a growth defect when single amino acids were present in the medium, such as leucine, phenylalanine, valine, or methionine, and the *gap2Δ/gap2Δ* mutant showed a lower transport capacity for certain amino acids. The other single mutants did not show any major defect in growth or transport capacities, indicating that they probably share redundant substrate specificities or that these transporters are not expressed or are not localized to the plasma membrane under the experimental conditions. The results obtained with the GFP-tagged genes (Fig. 5) confirmed that these genes are differentially expressed and/or localized, depending on the environmental conditions. This suggests that in the human body, the different *GAP* genes may be differentially expressed depending on the location. This would not be surprising, as *Candida* colonizes many different niches in and on the body and can live in several niches both commensally and as a pathogen.

In *S. cerevisiae*, *Sc*Gap1 is not only an amino acid transporter but also a sensor for rapid activation of the PKA pathway when nitrogen-starved cells are provided with amino acids (19, 47). We previously showed that expression of three *C. albicans* Gap permeases (Gap1, Gap2, and Gap6) in *S. cerevisiae* resulted in activation of trehalase, the PKA signaling pathway target, upon addition of specific amino acids to nitrogen-starved *S. cerevisiae* cells (21). However, in *C. albicans* we are not able to show an effect of specific amino acid transporters on the activation of trehalase. We previously showed that addition of glucose to glucose-starved cells also did not result in activation of trehalase (48). It is still possible that some of the Gap proteins in *Candida* activate the PKA pathway, but we are currently unable to prove this.

Real-time PCR analysis and GFP-protein fusion constructs showed a clearly distinct expression pattern between *GAP2* and the five other *GAP* permease genes. *GAP2* is not expressed in rich medium, but upon transfer of cells into nitrogen starvation medium expression is strongly induced, and the protein is plasma membrane localized. Maximal membrane localization persists only for a few hours, as longer incubation periods showed the GFP signal in intracellular vesicles and no longer localized to the membrane. This is clearly different from *S. cerevisiae*. The other Gap permeases are expressed and plasma membrane localized when cells are growing in rich medium. Transfer to nitrogen starvation medium results in their relocalization to the vacuole or intracellular vesicles. They may undergo a complicated regulation under different specific conditions in the host.

In *C. albicans*, there is a direct connection between the sensing of amino acids by the Csy1 sensor and the expression of *SAP2*, which encodes a secreted aspartyl proteinase, and the expression of Gap2. This is because the transcription factors Stp1 and Stp2 are proteolytically activated upon sensing of amino acids by Csy1 and they subsequently induce the expression of *SAP2* and *GAP*2, respectively (49). The GATA transcription factors Gln3 and Gat1, which are involved in nitrogen catabolite repression, are required for the expression of *STP1* when proteins are the only nitrogen source available (50, 51). Possibly, *SAP2* must first be expressed before the *GAP* genes are induced, and that is why they are only transiently localized at the plasma membrane. Whereas *S. cerevisiae* lives in environments with fluctuating concentrations of nitrogen sources, *C. albicans* lives in a more stable environment in the host, whose cells also require nitrogen, so the rapid signaling pathways reacting to the presence of nitrogen sources might not be mediated by activation of the PKA pathway but rather as the result of normal metabolism.

An interesting finding was the role of Gap4 in the uptake of SAM. Based on the heterologous expression of *GAP4* in a yeast *sam3* mutant, we found that Gap4 is a SAM transporter. Moreover, deletion of *GAP4* results in a complete defect in SAM-induced morphogenesis. Both results suggest that Gap4 transports SAM, which is subsequently used inside the cells to trigger morphogenesis. When we deleted *GAP4*, SAM transport was only reduced by about 20% compared to that of the wild-type strain (data not shown). This suggests that other transporters may be the reason why transport is only reduced by 20%. Alternatively, it is possible that a small reduction in SAM transport strongly affects a metabolic or signal transduction pathway involved in morphogenesis. We are currently further investigating SAM-induced morphogenesis in *C. albicans*. SAM is known to function as a methyl donor, and therefore it may affect methylation of DNA, histones, or other proteins that may result in the yeast-to-hypha transition. Whereas for *C. albicans* there are few data, for *S. cerevisiae* results have been obtained that clearly link histone methylation with pseudohyphal growth (52). Previously, we showed that methionine is a strong inducer of morphogenesis (38). SAM is also known as an acyl acceptor, where an acyl group is placed on the methionyl amine of SAM, a reaction that results in the production of homoserine lactones, which are important quorum-sensing molecules in some bacteria (53). So far, a role of SAM regarding quorum sensing in *C. albicans* or, for example, during mixed bacterium-fungus biofilm infections, has not been studied. As SAM is a bacterial product and SAM induces morphogenesis in *C. albicans*, it is possible that Gap4 may be an interesting protein that is involved in mixed infections. It will also be interesting to see in the future what the role of the different Gap permeases may be under *in vivo* conditions. We already generated a triple deletion strain, but it would be interesting to have strains where only one of the six permeases is present. With the use of CRISPR-Cas9, generating such strains may be more convenient compared to the current classical gene disruption strategies.

## MATERIALS AND METHODS

### Sequence analysis.

For BLAST analysis, protein sequences from the *C. albicans* database (CGD; http://www.candidagenome.org/) and the *S. cerevisiae* database (SGD; http://www.yeastgenome.org/) were used. Phylogenetic analysis of all known amino acid permeases in *S. cerevisiae*, the six-member *Ca*Gap family, and all other known amino acid permeases in *C. albicans* was performed by using of the MEGA software (Molecular Evolutionary Genetics Analysis; version 4), using the neighbor-joining method (54) with 500 bootstrap replicates (the bootstrap consensus tree was inferred from 500 replicates). Distances between the permease names are related to the degree of divergence between sequences.

### Strains and growth conditions.

All strains used in this work are listed in Table 2. All strains were routinely grown in YPD medium (1% yeast extract, 2% peptone, 2% glucose) or in YNB medium (0.17% YNB without amino acids and without ammonium sulfate) supplemented when indicated with BSM (Brent supplement mixture) and/or with 5 mg/ml of ammonium sulfate, 300 µg/ml of ammonium sulfate, or single amino acids as the sole source of nitrogen. For transport experiments or for GFP fluorescence microscopy experiments, strains were starved of nitrogen (NS; 0.17% YNB without amino acids and without ammonium sulfate). All media were supplemented when indicated with 2% glucose, except that nitrogen starvation medium was supplemented with 4% glucose and SLD medium was supplemented with 0.1% glucose. Solid media were supplemented with 2% agar. For induction of morphology, different hypha-inducing media were used: Spider medium (1% nutrient broth, 0.2% K_2_HPO_4_, 1.35% agar, and 1% mannitol as a carbon source; pH 7.2) (42); Lee medium (described previously [41]); YNB with GlcNAc (0.17% YNB without amino acids or ammonium sulfate and with 100 µg/ml *N*-acetylglucosamine); synthetic low ammonium (SLAD) medium (0.17% YNB without amino acids or ammonium sulfate and with 50 µM ammonium sulfate and 2% glucose) (55); synthetic low-dextrose (SLD) medium (0.17% YNB without amino acids or ammonium sulfate and with 5 mg/ml of ammonium sulfate and 0.1% glucose; pH adjusted to 6.5) (38).

**TABLE 2:** *C. albicans* and *S. cerevisiae* strains used in this study

Species and strain	Parental strain	Relevant genotype or characteristic(s)	Reference
*S. cerevisiae*			
GV287	JT 4500	Sigma 1278b *MATα*	This work
GV230	JT 4500	Sigma 1278b* MATα sam3*::*KanMX ura3-52*	This work
*C. albicans*			
SC5314		Wild-type strain	63
SN87	SC5314	*leu2Δ/leu2Δ his1Δ/his1Δ URA3/ura3*::*imm434 IRO1/iro1*::*imm434*	32
CAEB-5	BWP17	*ura3*::*imm434/ura3*::*imm434 HIS1*::*his1*::*hisG/his1*::*hisG arg4*::*hisG/arg4*::*hisG csy1*::*ARG4/csy1*::*URA3*	25
BSC1	SN87	*LEU2/leu2Δ HIS1/his1Δ URA3/ura3*::*imm434 IRO1/iro1*::*imm434*	This study
LK1H (*gap1/GAP1*)	SN87	*leu2Δ/leu2Δ his1Δ/his1Δ URA3/ura3*::*imm434 IRO1/iro1*::*imm434 GAP1/gap1::CmLEU2*	This study
LK1 (*gap1*)	LK1H	*leu2Δ/leu2Δ his1Δ/his1Δ URA3/ura3*::*imm434 IRO1/iro1*::*imm434 gap1*::*CdHIS1/gap1*::*CmLEU2*	This study
LK2H (*gap2/GAP2*)	SN87	*leu2Δ/leu2Δ his1Δ/his1Δ URA3/ura3*::*imm434 IRO1/iro1*::*imm434 GAP2/gap2*::*CmLEU2*	This study
LK2 (gap2)	LK2H	*leu2Δ/leu2Δ his1Δ/his1Δ URA3/ura3*::*imm434 IRO1/iro1*::*imm434 gap2*::*CdHIS1/gap2*::*CmLEU2*	This study
LK3H (*gap3/GAP3*)	SN87	*leu2Δ/leu2Δ his1Δ/his1Δ URA3/ura3*::*imm434 IRO1/iro1*::*imm434 GAP3/gap3*::*CmLEU2*	This study
LK3 (*gap3*)	LK3H	*leu2Δ/leu2Δ his1Δ/his1Δ URA3/ura3*::*imm434 IRO1/iro1*::*imm434 gap3*::*CdHIS1/gap3*::*CmLEU2*	This study
LK4H (*gap4/GAP4*)	SN87	*leu2Δ/leu2Δ his1Δ/his1Δ URA3/ura3*::*imm434 IRO1/iro1*::*imm434 GAP4/gap4*::*CmLEU2*	This study
LK4 (*gap4*)	LK4H	*leu2Δ/leu2Δ his1Δ/his1Δ URA3/ura3*::*imm434 IRO1/iro1*::*imm434 gap4*::*CdHIS1/gap4*::*CmLEU2*	This study
LK5H (*gap5/GAP5*)	SN87	*leu2Δ/leu2Δ his1Δ/his1Δ URA3/ura3*::*imm434 IRO1/iro1*::*imm434 GAP5/gap5*::*CmLEU2*	This study
LK5 (*gap5*)	LK5H	*leu2Δ/leu2Δ his1Δ/his1Δ URA3/ura3*::*imm434 IRO1/iro1*::*imm434 gap5*::*CdHIS1/gap5*::*CmLEU2*	This study
LK6H (*gap6/GAP6*)	SN87	*leu2Δ/leu2Δ his1Δ/his1Δ URA3/ura3*::*imm434 IRO1/iro1*::*imm434 GAP6/gap6*::*CmLEU2*	This study
LK6 (*gap6*)	SN87	*leu2Δ/leu2Δ his1Δ/his1Δ URA3/ura3*::*imm434 IRO1/iro1*::*imm434 gap6*::*CdHIS1/gap6*::*CmLEU2*	This study
LK12 (*gap1*/2)	*gap1*	*leu2Δ/leu2Δ his1Δ/his1Δ URA3/ura3*::*imm434 IRO1/iro1*::*imm434 gap1*::*CdHIS1/gap1*::*CmLEU2 gap2*::*FRT/gap2*::*FRT*	This study
LK16 (*gap1*/6)	*gap1*	*leu2Δ/leu2Δ his1Δ/his1Δ URA3/ura3*::*imm434 IRO1/iro1*::*imm434 gap1*::*CdHIS1/gap1*::*CmLEU2 gap6*::*FRT/gap6*::*FRT*	This study
LK26 (*gap2/6*)	*gap2*	*leu2Δ/leu2Δ his1Δ/his1Δ URA3/ura3*::*imm434 IRO1/iro1*::*imm434 gap2*::*CdHIS1/gap2*::*CmLEU2 gap6*::*FRT/gap6*::*FRT*	This study
LK126 (*gap1/2/6*)	*gap1/2*	*leu2Δ/leu2Δ his1Δ/his1Δ URA3/ura3*::*imm434 IRO1/iro1*::*imm434 gap1*::*CdHIS1/gap1*::*CmLEU2 gap2*::*FRT/gap2*::*FRT gap6*::*FRT/gap6*::*FRT*	This study
LK2R (*GAP2-Ri*)	*gap2*	*leu2Δ/leu2Δ his1Δ/his1Δ URA3/ura3::imm434 IRO1/iro1::imm434 gap2::GAP2::FRT::CdHIS1/gap2::CmLEU2*	This study
LK4R (*GAP4-Ri*)	*gap4*	*leu2Δ/leu2Δ his1Δ/his1Δ URA3/ura3*::*imm434 IRO1/iro1*::*imm434 gap4*::*GAP4*::*FRT*::*CdHIS1/gap4*::*CmLEU2*	This study
LK1HG *GAP1-GFP*	LK1H	*leu2Δ/leu2Δ his1Δ/his1Δ URA3/ura3*::*imm434 IRO1/iro1*::*imm434 gap1*::*GAP1- GFP*::*CaHIS1/gap1*::*CmLEU2*	This study
LK2HG *GAP2-GFP*	LK2H	*leu2Δ/leu2Δ his1Δ/his1Δ URA3/ura3*::*imm434 IRO1/iro1*::*imm434 gap2*::*GAP2-GFP*::*CaHIS1/gap2*::*CmLEU2*	This study
LK3HG *GAP3-GFP*	LK3H	*leu2Δ/leu2Δ his1Δ/his1Δ URA3/ura3*::*imm434 IRO1/iro1*::*imm434 gap3*::*GAP3-GFP*::*CaHIS1/gap3*::*CmLEU2*	This study
LK4HG *GAP4-GFP*	LK4H	*leu2Δ/leu2Δ his1Δ/his1Δ URA3/ura3*::*imm434 IRO1/iro1*::*imm434 gap4*::*GAP4-GFP*::*CaHIS1/gap4*::*CmLEU2*	This study
LK5HG *GAP5-GFP*	LK5H	*leu2Δ/leu2Δ his1Δ/his1Δ URA3/ura3*::*imm434 IRO1/iro1*:*:imm434 gap5*::*GAP5-GFP*::*CaHIS1/gap5*::*CmLEU2*	This study
LK6HG *GAP6-GFP*	LK6H	*leu2Δ/leu2Δ his1Δ/his1Δ URA3/ura3*::*imm434 IRO1/iro1*::*imm434 gap6*::*GAP6-GFP::CaHIS1/gap6*::*CmLEU2*	This study

### Deletion and reintegration.

We generated single *GAP* deletion strains by using the fusion PCR method (31, 32) (see Table 2 for the different strains that were obtained). The SN87 strain was used as a starting strain. We also generated double and triple *GAP* deletion strains: *gap1Δ/gap1Δ gap2Δ/gap2Δ*, *gap2Δ/gap2Δ gap6Δ/gap6Δ*, *gap1Δ/gap1Δ gap6Δ/gap6Δ*, and *gap1Δ/gap1Δ gap2Δ/gap2Δ gap6Δ/gap6Δ*, using the *SAT1* flipper cassette (32, 33). Single *GAP* deletion strains created with the fusion PCR method were used as starting strains. For creation of *CaGAP2* and *CaGAP4* reintegrants, the pSFS2A plasmid with the *SAT1* cassette was used (34), starting with the single *GAP* deletion strains *gap2Δ/gap2Δ* and *gap4Δ/gap4Δ*. The *CaGAP2* or the* CaGAP4* gene with approximately 500 bp of promoter sequence and approximately 500 bp of terminator sequence was integrated between the KpnI and the XhoI restriction sites of the pSFS2A plasmid, and the *HIS1* marker was integrated between the NotI and the SacII restriction sites of the pSFS2A plasmid. The reintegration cassette was cut out of the plasmid with KpnI and SacII and used for homologous recombination into the *gap2Δ/gap2Δ* and* gap4Δ/gap4Δ* strains (*Candida maltosa LEU2* and *Candida dubliniensis HIS1* are present at the loci of the *GAP* alleles). Reintegrants were selected on YPD medium plus 200 µg/ml nourseotricin, and afterwards the *SAT1* cassette was removed as described elsewhere (34). The reintegrants were verified by PCR. All primers used for construction and diagnosis of reintegrants are listed in Table S1 in the supplemental material.

10.1128/mSphere.00284-16.3 Table S1  List of oligonucleotide sequences. Download Table S1, XLSX file, 0.01 MB. Copyright © 2016 Kraidlova et al. 2016 Kraidlova et al. This content is distributed under the terms of the Creative Commons Attribution 4.0 International license.

### Growth curve measurements.

To test the growth capacities of our strains, a microplate reader technique was applied (56). Freshly grown cells (on YPD plates) were suspended in sterile PBS (phosphate-buffered saline: 0.8 g/liter NaCl, 0.2 g/liter KCl, 1.44 g/liter Na_2_HPO_4_, 0.24 g/liter KH_2_PO_4_; pH 7.4) to obtain an optical density at 600 nm (OD_600_) of 1 and used to inoculate liquid YNB medium (supplemented with 300 µg/ml of ammonium sulfate or single amino acids as sole sources of nitrogen, unless otherwise indicated) and grown to an OD_600_ of 0.002. Aliquots (100 μl) were transferred to a 96-well plate. The cell cultures in the plate were incubated at 30°C in an ELx808 absorbance microplate reader, and the optical density in each well was measured at 595 nm over a period of 24 h under continuous shaking. Growth curve measurements were repeated at least twice, each time with four identical parallel cultures for each strain and each set of tested conditions.

### Growth assays.

The growth rate in the presence of different nitrogen sources was assayed by a classical drop test. Freshly grown cells (on YPD plates) of each tested strain were suspended in sterile PBS and adjusted to the same initial OD_600_ of 1. Tenfold serial dilutions were prepared, and 3-µl aliquots were spotted on a series of plates containing YNB with 300 µg/ml of ammonium sulfate or a single amino acid as sole source of nitrogen (unless otherwise indicated). Plates were incubated at 30°C for 3 days, and digital gray-scale images of growing colonies were obtained using a Nicon Coolpix4500 digital camera. Representative results are reported.

### Measurement of transport activity.

*Candida* cells for amino acid uptake measurements were grown to exponential phase in liquid YNB medium supplemented with BSM and ammonium sulfate, harvested by centrifugation, resuspended in nitrogen starvation medium, and further cultivated either for 24 h for nitrogen-poor condition transport experiments or for only 12 h and then harvested again by centrifugation and resuspended in YNB supplemented with BSM and 5 mg/ml of ammonium sulfate. The second type of cultures were grown for another 5 to 6 h for nitrogen-rich condition transport experiments. *S. cerevisiae* cells for SAM uptake measurements were grown in complete synthetic medium (MP Biomedicals) at 30°C overnight, then diluted in YNB medium (5 mg/ml ammonium sulfate) and grown at 30°C overnight.

Cells for amino acid or SAM uptake were cooled on ice for 20 min, harvested by centrifugation, washed with 5 ml 10 mM morpholineethanesulfonic acid (MES) buffer (pH 6.5; containing 0.1 mM EDTA for transport of *Candida*), and resuspended at a cell density of 80 mg (wet weight)/ml in corresponding fresh medium. Thereafter, 40 µl of the cell suspension was preincubated at 30°C in a shaking water bath for 10 min, then 10 µl of a mixture of unlabeled (2 mM final concentration for amino acid transport and 0.1 mM for SAM transport) and ^14^C-labeled l-amino acid (2 mM unlabeled amino acid [final concentration] plus ^14^C-labeled l-amino acid, to obtain a specific activity of 3,000 cpm/nmol) or ^3^H-labeled *S*-adenosyl methionine (0.1 mM unlabeled SAM [final concentration] plus ^3^H-labeled SAM, to obtain a specific activity of 3,000 cpm/nmol) was added to the cells, and the resulting mixture was incubated at 30°C for 1 min. The reaction was stopped by cooling via addition of 5 ml ice-cold water. The obtained suspension was filtered through a glass microfiber filter (Whatman GF/C; retention particle size, 1.2 µm) prewet with 2 mM unlabeled amino acid or 0.1 mM unlabeled SAM and immediately washed twice with 4 ml ice-cold water. Afterwards, the filter was deposited in a scintillation tube which contained 4 ml scintillation liquid (Lumagel Safe; PerkinElmer). Radioactivity was measured in a liquid scintillation counter (model LS6500; Beckman Coulter, Inc.). For each determination, three samples and two blanks were taken (addition of ice-cold water followed by addition of the labeled amino acid). In addition, 500 µl of the cell suspension was used to determine the protein content based on the Bradford method (57). The labeled amino acid or SAM solution in a total volume of 10 µl was used for the determination of specific activity. The transport activity was calculated from the measured radioactivity and estimated protein content in the samples, and it was expressed as nanomoles transported per minute per milligram of protein. Three independent experiments were performed, and either the standard deviation was calculated between these experiments (for amino acid transport assay) or between different transformants in one representative experiment (for SAM transport in *S. cerevisiae* assay). A two-sided *t* test was used to determine statistical significance between transport rates of different strains.

### RNA extraction and quantitative real-time PCR.

Cells for RNA extraction were grown to exponential phase in liquid YPD medium, harvested by centrifugation, and either (i) resuspended in nitrogen starvation medium and samples taken after 15 or 30 min or (ii) resuspended in nitrogen starvation medium, cultivated for a further 12 h, harvested by centrifugation, and resuspended in YPD or YNB with 5 mg/ml of ammonium sulfate supplemented with 100 µg/ml single amino acid (for *csy1* qRT-PCR experiments), and samples were taken after 15 or 30 min. The samples were cooled by addition of ice-cold water, centrifuged at 4°C, and washed once with 1 ml ice-cold water, and the pellet was flash-frozen in liquid nitrogen and stored at −80°C. Total RNA was extracted from yeast cells with Trizol reagent (Invitrogen). RNA purity was determined with a NanoDrop Technologies ND-1000 spectrophotometer. After digestion with RNase-free DNase (Promega) to eliminate genomic contamination, cDNA was synthesized from 1 µg of total RNA with the Bio-Rad iScript cDNA synthesis kit according to the manufacturer’s instructions. The relative gene expression was determined by quantitative PCR on a StepOnePlus real-time PCR system (Applied Biosystems) using the KAPA fast kit (KAPA Biosystems). This kit uses SYBR green, which is a fluorogenic dye that shows little fluorescence in solution but emits a strong fluorescent signal upon binding to double-stranded DNA, resulting in increased fluorescence as the PCR product accumulates. The fold change was calculated using the comparative threshold cycle (*C*_*T*_) method, and error bars showing the range of possible relative quantity values were defined by the standard error of the ΔΔ*C*_*T*_ values (see http://www.science.smith.edu/cmbs/wp-content/uploads/sites/36/2015/09/Analyzing-your-QRT-for-relative-2%5E-%E2%88%86%E2%88%86Ct.pdf). (58, 59). All data were normalized to *TEF1*, which was used as the reference housekeeping gene. The data presented in Fig. 4 are the summary of three independent experiments. The sequences of the primers used in this assay are provided in Table S1 in the supplemental material.

### GAPx-GFP strain construction.

To tag *GAP* genes with *GFP* (optimized for *C. albicans* [60]), *GAP* gene-specific sequences (approximately 70 nucleotides in length) were added to the universal primer sequences (Table S1; lowercase and uppercase letters, respectively) in the p*GFP-HIS1* plasmid (61). *CaGAPx-GFP* strains were created from *gapx/GAPx* His^−^ heterozygous strains (where only one copy of *CaGAP* is still present) by homologous recombination of *GFP* sequences into the 3′ end of the *CaGAPs* open reading frames. *CaGAP-GFP* mutants were selected by growth on medium without histidine and verified by PCR afterwards (see Table S1 for a list of the primers used).

### Fluorescence microscopy.

Cells for fluorescence microscopy were grown to exponential phase in YNB medium supplemented with 5 mg/ml of ammonium sulfate, harvested by centrifugation, and either (i) resuspended in nitrogen starvation medium and further cultivated for 1, 3, or 12 h, or (ii) resuspended in nitrogen starvation medium, further cultivated for 12 h, harvested by centrifugation, and resuspended in YPD or YNB supplemented with ammonium sulfate (5 mg/ml), grown for another 6 or 18 h, resuspended in YNB medium supplemented with a single amino acid (100 µg/ml) as the nitrogen source, and grown for another 5 to 6 h. Nitrogen-starved cells grown for 1, 3, 5 to 6, 12, or 18 h were collected by centrifugation and used directly without fixation, visualized by fluorescence microscopy (the fluorescence signal in the cells was observed under an Olympus AX70 microscope using a U-MWB cube with a 450- to 480-nm excitation filter and 15-nm barrier filter) and differential interference contrast (DIC) optics (Nomarski). Various exposure times for different media were used (as indicated in the figure legends). Multiple images of representative cells (almost the whole population behaved similarly under our conditions) were captured with a DP70 digital camera using the program DP Controller. Subsequent processing was done using Adobe Photoshop.

### Colony morphology.

To determine the ability to undergo the yeast-to-hypha transition, freshly grown *C. albicans* cells (on YPD plates) were suspended in sterile PBS to obtain an OD_600_ of 0.1. This suspension was diluted 1/1,0000 in four steps, leading to a cell suspension containing approximately 100 cells/ml. Afterwards, 100 µl of the suspensions was plated on the indicated media. In some cases, SLD and SLAD media were supplemented with 5 µg/ml of methionine or SAM. Plates were incubated at 30°C or 37°C. Pictures of representative colonies were taken using a binocular microscope.

### Complementation of *ScSAM3* with *GAP4*.

The *ScSAM3* construct with restriction sites (PstI and BamHI) was made through PCR of 1,000 bp of promoter sequence, the *ScSAM3* gene, and 500 bp of terminator sequence by using the primers SAM3promF-PstI and SAM3terR-BamHI.

The *CaGAP4* construct was made by fusion PCR between the 1,000-bp *ScSAM3* promoter, the *CaGAP4* gene, and the 500-bp *ScSAM3* terminator. To make this construct, the following primers were used: SAM3promF-PstI, SAM3promR-fusion, GAP4F-fusion, GAP4Revfusion, SAM3terR-BamHI, and SAM3terF-fusion. Both constructs were ligated into the multiple cloning site of the YCplac33 vector upon restriction digestion with PstI and BamHI. The *S. cerevisiae sam3Δ* strain GV230 was transformed with the newly created plasmids and empty YCplac33 vector, using the Gietz transformation method (62). Three of the transformants and the wild-type strain GV287 were grown in 50 ml of YNB plus 5 mg/ml ammonium sulfate medium at 30°C overnight. Afterwards, the cultures were centrifuged and cells were washed with 5 ml MES buffer (pH 6). The pellet was resuspended in YNB plus ammonium sulfate medium to achieve a concentration of 80 mg/ml. The transport experiment was carried out as stated above (SAM concentration, 0.5 mM).

### Reproducibility of results.

All experiments were performed at least three times with independent deletion strains and/or cultures. The results always showed consistent trends, i.e., differences between strains and mutants were highly reproducible.

The results of single time point measurements (e.g., uptake rate of amino acid, mRNA expression levels) are based on three independent measurements, and standard deviations are indicated by the error bars in the graphs in the figures.
