# Supplementary figures and images for: Characterization of the Candida albicans Amino Acid Permease Family: Gap2 Is the Only General Amino Acid Permease and Gap4 Is an S-Adenosylmethionine (SAM) Transporter Required for SAM-Induced Morphogenesis
Source: mSphere. 2016 Dec 21;1(6):e00284-16. doi: 10.1128/mSphere.00284-16 (PMC5177730; doi:10.1128/mSphere.00284-16)

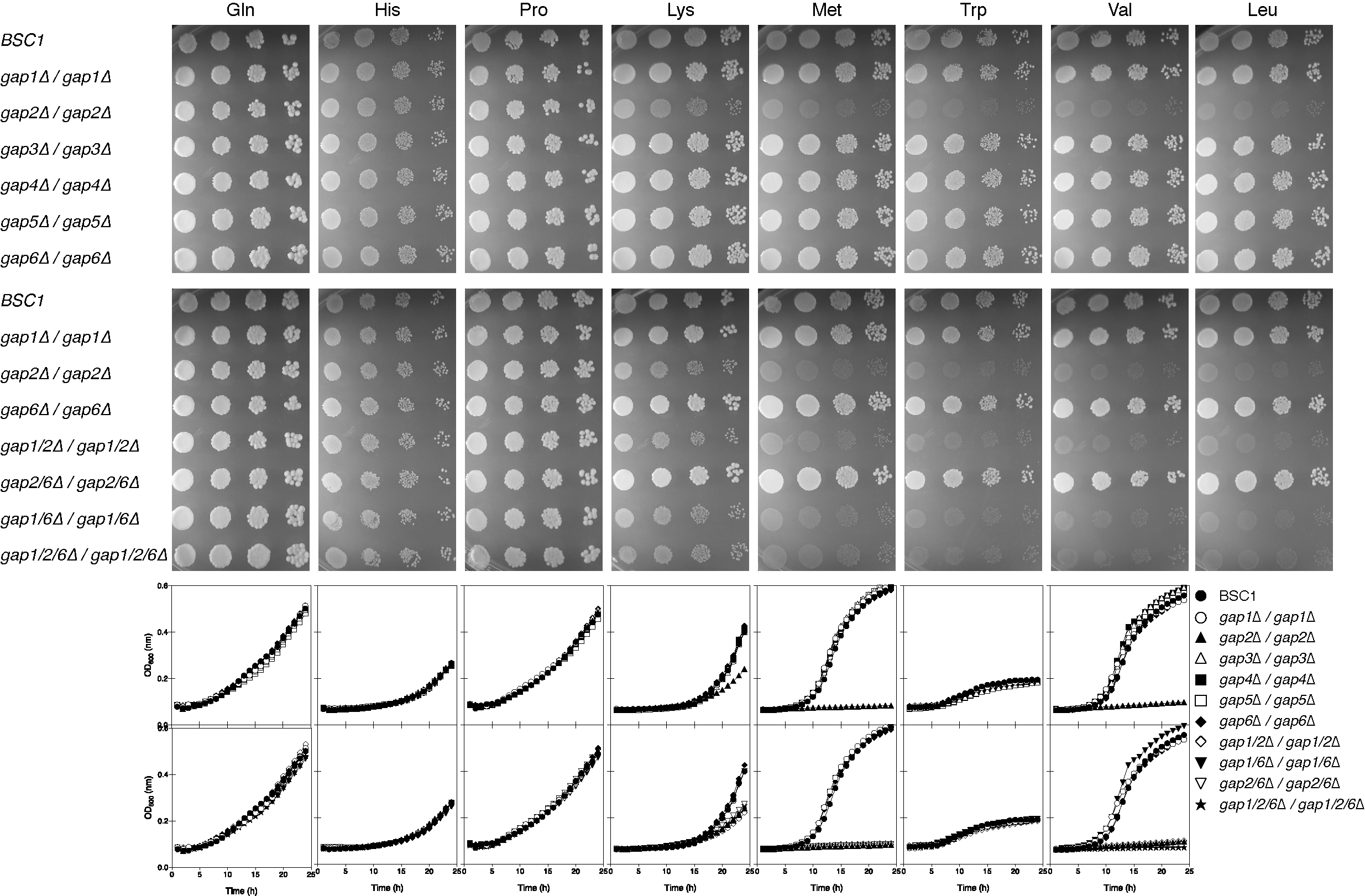

Supplement: Figure S1 [file sph006162210sf1.tif]

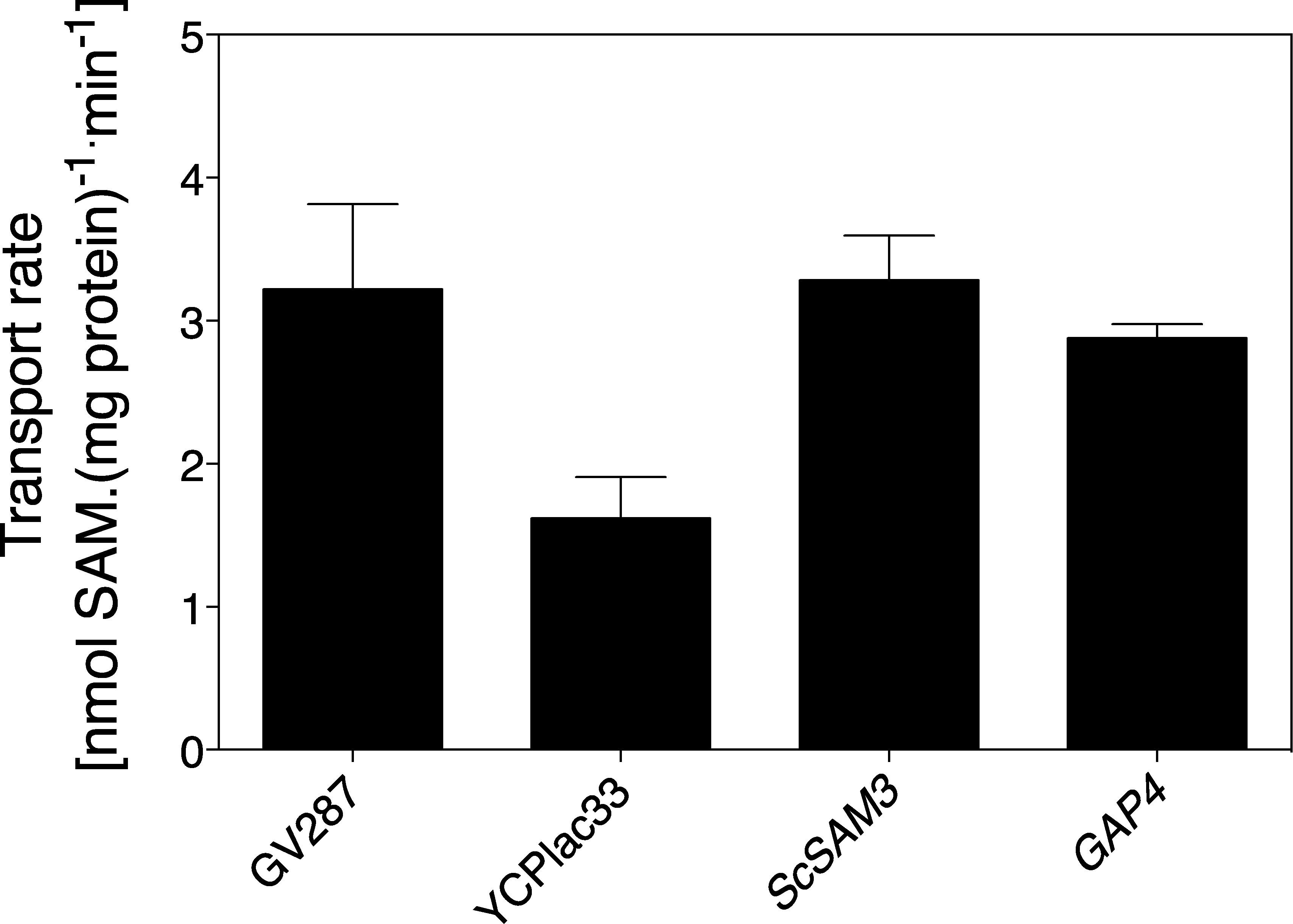

Supplement: Figure S2 [file sph006162210sf2.tif]
